# Robot-assisted surgery for rectal cancer with tumor-associated dermatomyositis during preoperative chemotherapy: A case report and literature review

**DOI:** 10.1097/MD.0000000000043758

**Published:** 2025-08-08

**Authors:** Eiho Sawamura, Hiroki Hamamoto, Yusuke Suzuki, Toru Kuramoto, Takafumi Shima, Kazuya Kitada, Mitsuhiro Asakuma, Sang-Woong Lee

**Affiliations:** a Department of General and Gastroenterological Surgery, Osaka Medical and Pharmaceutical University, Takatsuki, Japan.

**Keywords:** case report, dermatomyositis, paraneoplastic syndrome, rectal cancer, robotic-assisted surgery, transcriptional intermediary factor 1-gamma antibody

## Abstract

**Rationale:**

Dermatomyositis (DM) is a systemic inflammatory disorder involving the muscles and skin and is often associated with malignancies in adults. Anti-transcriptional intermediary factor 1-gamma (anti-TIF1-γ) antibodies are specific markers of a subset of DM with a high risk of cancer, highlighting the importance of recognizing this paraneoplastic syndrome for early diagnosis and treatment. This report describes a rare case of tumor-associated DM that developed during chemotherapy for advanced rectal cancer and emphasizes the importance of multidisciplinary management in such conditions.

**Patient concerns:**

A 75-year-old woman presented with rectal bleeding and progressive symptoms, including severe erythema, rash, muscle weakness, and general fatigue, following preoperative chemotherapy for rectal cancer. The symptoms severely impacted her quality of life, raising concerns about a potential paraneoplastic syndrome.

**Diagnoses:**

The patient was diagnosed with tumor-associated DM based on clinical findings of a heliotrope rash, Gottron’s sign, and elevated muscle enzyme levels. The presence of anti-TIF1-γ antibodies confirmed the diagnosis. Preoperative evaluation identified rectal cancer as stage IIa (pT3, N0, M0) and the likely underlying malignancy contributing to DM.

**Interventions:**

The patient underwent immunotherapy with steroids and high-dose intravenous immunoglobulin, which stabilized her condition. Subsequently, a robot-assisted Hartmann’s operation was performed to resect the rectal tumor and establish a colostomy. Postoperative care included monitoring for DM symptom resolution and managing her nutritional and rehabilitation needs.

**Outcomes:**

Within 3 weeks of surgery, the patient’s muscle enzyme levels normalized, and her symptoms, including erythema and muscle weakness, resolved. Follow-up evaluations showed no recurrence of DM or cancer over the subsequent year, indicating a successful outcome with a multidisciplinary approach.

**Lessons:**

This case underscores the importance of early recognition and management of paraneoplastic syndromes such as tumor-associated DM. A multidisciplinary approach combining immunotherapy and surgical intervention for the underlying malignancy can lead to favorable outcomes. This case also highlights the significance of long-term monitoring for both cancer recurrence and autoimmune complications in such patients.

## 1. Introduction

Dermatomyositis (DM) is a systemic inflammatory disorder that affects the muscles and skin and is frequently associated with malignancies in adults. Anti-transcriptional intermediary factor 1-gamma (anti-TIF1-γ) antibodies are specific to a subset of DM associated with a high risk of cancer. Recognizing this paraneoplastic syndrome is crucial for its timely diagnosis and treatment. In this report, we describe a rare case of tumor-associated DM that developed during chemotherapy for advanced rectal cancer. This case report has been reported in line with the SCARE criteria.

Paraneoplastic DM accounts for approximately 10% to 15% of adult-onset DM cases.^[1–3]^ Cancers most associated with DM include gastric, lung, pancreatic, and ovarian cancers; however, the association with rectal cancer is relatively rare, making such cases noteworthy.

This case is unique because the onset of DM occurred during neoadjuvant chemotherapy, rather than preceding or at the time of cancer diagnosis. This temporal relationship raises important clinical questions about the immunological impact of chemotherapy and its potential role in triggering paraneoplastic syndromes. The diagnostic and therapeutic implications of identifying anti-TIF1-γ antibodies in patients presenting with DM are substantial, as it necessitates thorough malignancy screening and may influence the timing and modality of cancer treatment.

This case report was prepared in accordance with the CARE Checklist.

## 2. Case report

**Figure 1.**
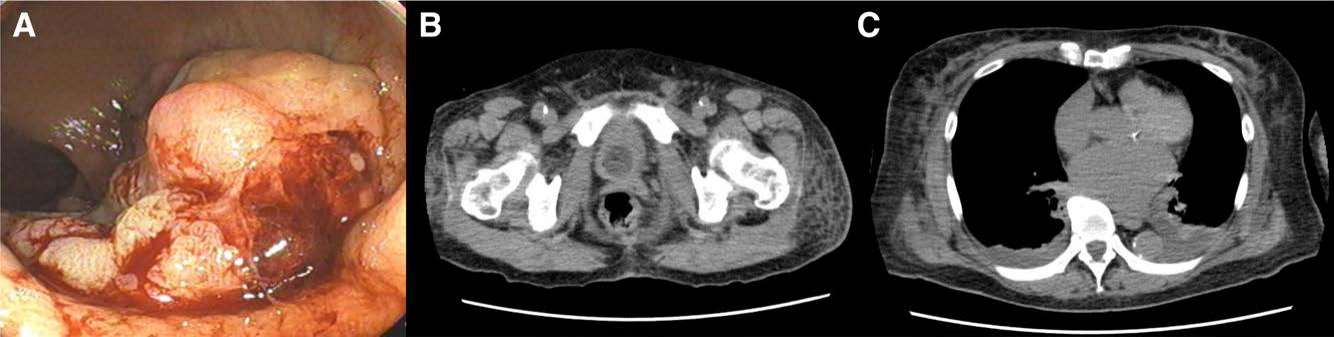
Representative images. (A) Colonoscopy image showing a type 2 tumor in the lower rectum. (B) Computed tomography scan reveals rectal wall thickening with contrast enhancement. (C) Bilateral pleural effusion is observed.

**Figure 2.**
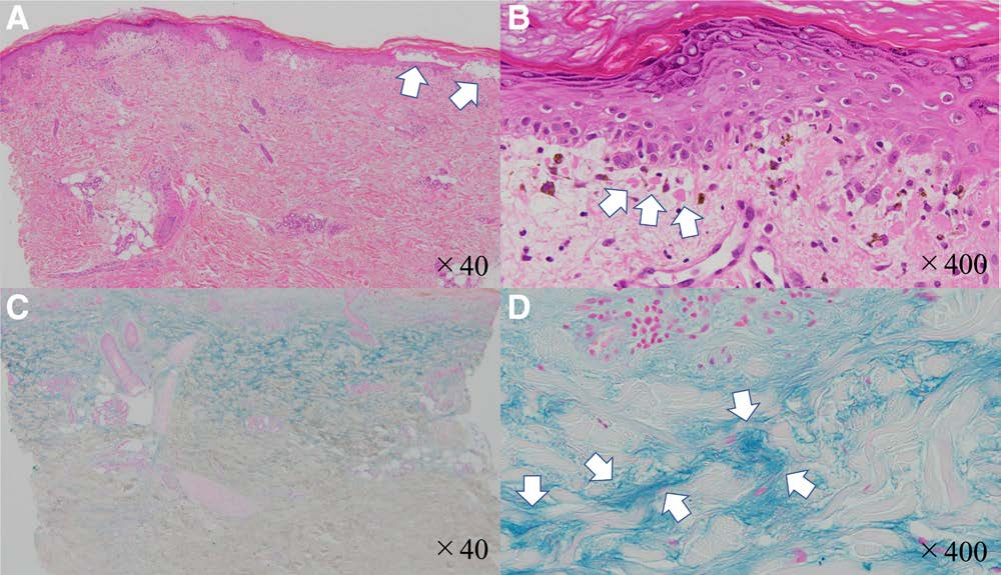
Skin biopsy. (A) H&E stain, ×40, blister formation observed at the dermo-epidermal junction (white arrow). (B) H&E stain, ×400, vacuolar degeneration accompanied by numerous Civatte bodies (white arrow). (C) Alcian blue staining, ×40. (D) Alcian blue staining, ×400, mucin deposition observed in the dermis tissue (white arrow). H&E = hematoxylin and eosin.

**Figure 3.**
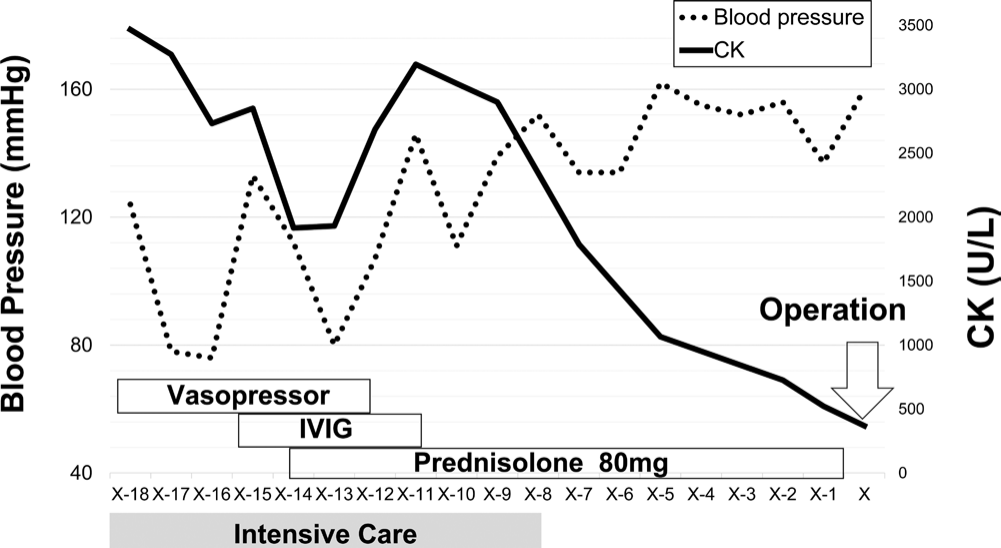
Progress following hospitalization. CK = creatine kinase, IVIG = intravenous immunoglobulin.

**Figure 4.**
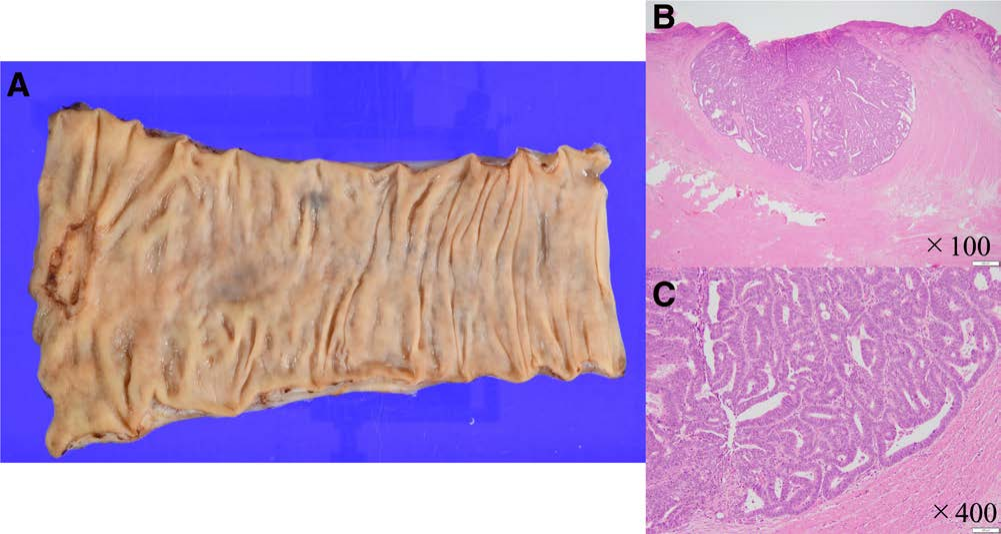
Histopathological finding. (A) Resected specimen. (B) H&E stain, ×100. (C) H&E stain, ×400, moderately differentiated tubular adenocarcinoma. H&E = hematoxylin and eosin.

A 75-year-old woman presented to her doctor with rectal bleeding. Colonoscopy and abdominal computed tomography revealed a type 2 lesion in the lower rectum (Fig. 1). There is no significant past medical or surgical history, drug or allergy history, nor family or social history to report. The patient received preoperative chemotherapy (FOLFOX [folinic acid, fluorouracil, and oxaliplatin]). During the second cycle of chemotherapy, the patient developed severe erythema, skin rash, muscle weakness, and general fatigue, which made walking difficult. She was admitted to our hospital and was diagnosed with tumor-associated DM. The diagnosis was based on the presence of a heliotrope rash, Gottron’s sign, elevated muscle enzymes, and positive anti-TIF1-γ antibody (Table 1). Regarding histological findings, characteristics of DM were observed on skin biopsy (Fig. 2). Treatment with steroids (prednisolone: 80 mg/d) and high-dose intravenous immunoglobulin (400 mg/kg/d) was initiated, and the patient’s condition was improved (Fig. 3). The patient subsequently underwent robot-assisted Hartmann’s operation. Intraoperatively, the tumor was resected and a colostomy was performed. The postoperative pathology report confirmed stage IIa rectal cancer (type 2, 1.5 cm × 2.5 cm, pT3, N0, M0; Fig. 4). Histopathological examination of the resected specimen revealed well-differentiated adenocarcinoma. The patient’s muscle enzyme levels normalized 3 weeks after surgery, and the symptoms of DM, including erythema, resolved. The patient showed no signs of recurrence or further symptoms of DM.

**Table 1:** Hematological, biochemical, and immunological laboratory findings.

Peripheral blood		
WBC	13,870	/μL
RBC	355 × 10^4^	/μL
Hb	10.1	g/dL
Plt	17.4 × 10^4^	/μL
Blood chemistry		
TP	4.5	g/dL
Alb	2.3	g/dL
AST	294	U/L
ALT	69	U/L
LDH	498	U/L
ALP	56	U/L
BUN	42	mg/dL
Cre	1.04	mg/dL
Na	138	mEq/L
K	4.6	mEq/L
Cl	107	mEq/L
CK	3467	U/L
BS	109	mg/dL
Ferritin	1067	ng/mL
CRP	2.32	mg/dL
Coagulation		
PT	14.4	s
PT-INR	1.16	
APTT	47.2	s
Myositis-specific autoantibodies		
TIF1-γ	(+)	
ARS	(−)	
Mi-2	(−)	
MDA5	(−)	

Alb = albumin, ALP = alkaline phosphatase, ALT = alanine aminotransferase, APTT = activated partial thromboplastin time, ARS = aminoacyl-tRNA synthetase, AST = aspartate aminotransferase, BS = blood sugar, BUN = blood urea nitrogen, CK = creatine kinase, Cre = creatinine, CRP = C-reactive protein, Hb = hemoglobin, LDH = lactate dehydrogenase, MDA5 = melanoma differentiation-associated gene 5, Mi-2 = anti-Mi-2 antibody, Plt = platelet, PT = prothrombin time, PT-INR = prothrombin time - international normalized ratio, RBC = red blood cell, TIF1-γ = transcriptional intermediary factor 1-gamma, TP = total protein, WBC = white blood cell.

## 3. Discussion

Here, we report a rare case of rectal cancer-associated DM that was successfully managed using a multidisciplinary approach. The patient’s overall condition improved with immunoglobulin and steroid therapy, followed by robot-assisted Hartmann’s surgery performed at the appropriate time. The patient received preoperative chemotherapy due to the advanced stage of rectal cancer (T3N0M0) and to attempt tumor downstaging. This is a standard treatment protocol for locally advanced rectal cancer, aiming to improve surgical outcomes and reduce the risk of recurrence. However, this chemotherapy was likely associated with the development of DM, which is a rare occurrence.

DM is an inflammatory myopathy of unknown etiology that involves skin lesions and muscle inflammation, particularly in the limbs. The characteristic skin symptoms include heliotrope rash (purple-red edema around the eyes) and Gottron’s papules (rash around small joints in the hands, elbows, and knees). These skin symptoms often precede muscle involvement and can persist even when muscle symptoms subside. Swallowing and speech difficulties may arise because of the involvement of the swallowing and articulation muscles, leading to dysphagia and dysarthria. Other systemic symptoms include interstitial pneumonia, arthritis, arrhythmia, vasculitis, and malignancy. In 1975, Bohan and Peter proposed the diagnostic criteria for DM, including symmetrical muscle weakness, elevated muscle enzymes, characteristic electromyography findings, muscle biopsy showing inflammation, and skin symptoms such as heliotrope rash and Gottron’s sign. In Japan, as of 2015, the diagnosis of DM was made if 4 or more of 9 specific criteria were met.

Treatment for DM generally includes corticosteroids, immunoglobulin therapy, and plasma exchange^[4]^; however, in cases of malignancy-associated DM, treating the underlying tumor is often crucial for improving the prognosis.^[5,6]^ This case demonstrates the effectiveness of treating malignancy to alleviate the symptoms of tumor-associated DM. Management of paraneoplastic DM requires prompt intervention for the underlying malignancy. In this case, tumor resection led to a significant improvement in DM symptoms, supporting existing evidence that effective oncologic control can ameliorate paraneoplastic manifestations.

The association between rectal cancer and DM is particularly rare, with only 18 cases reported between 1996 and 2024. Surgical treatment of rectal cancer with accompanying DM was performed in only 5 cases. Unlike most patients who are diagnosed with DM before undergoing cancer screening that subsequently identifies the tumor, our patient was initially diagnosed with rectal cancer and later developed DM during chemotherapy. At the time of rectal cancer diagnosis, no skin symptoms were present, but these symptoms emerged during chemotherapy, making this case distinct from the typical presentation. Surgical treatment of the tumor has been shown to improve DM symptoms in most cases.^[7]^ This further supports the idea that malignancy treatment is an effective approach for tumor-associated DM.^[8]^

DM exacerbation during preoperative chemotherapy for malignancies has previously been reported.^[9]^ These cases suggest that chemotherapy may trigger immune responses or muscle-related antigen-antibody reactions, resulting in DM flares. However, as in our case, surgery to remove the tumor can lead to the resolution of the symptoms of DM.

It is important to note that DM associated with the anti-TIF1-γ antibody, present in 20% of adult cases, is particularly linked to malignancies.^[10]^ In cases with positive anti-TIF1-γ, 60% to 90% of patients also have cancer.^[11–13]^ Additionally, these patients may present with dysphagia, myositis, or elevated ferritin levels.^[14]^ In such cases, comprehensive cancer screening is critical even if no malignancy is detected at the onset of DM.^[15]^ Close monitoring for at least 3 years is recommended to identify any developing tumors.^[14]^

In this case, the patient experienced preoperative dysphagia, requiring enterostomy during the surgery to initiate early nutritional therapy. Dysphagia is common in anti-TIF1-γ-positive DM,^[16–18]^ and early consideration of enteral nutrition is advisable.^[19]^ Inflammatory cell infiltration and muscle atrophy have been observed in pathological studies, and the treatment typically includes pharmacotherapy, swallowing rehabilitation, and sometimes surgery.

## 4. Conclusion

This case highlights the successful management of rectal cancer-associated DM using a multidisciplinary approach, including timely surgery and immunotherapy with steroids and intravenous immunoglobulins. DM associated with anti-TIF1-γ antibodies presents a high risk of malignancy, requiring comprehensive cancer screening and long-term monitoring. The patient’s symptoms, including muscle weakness and skin lesions, resolved after tumor resection. This emphasizes the critical role of treating the underlying malignancy in alleviating paraneoplastic symptoms. This report underscores the importance of early diagnosis, appropriate therapeutic intervention, and multidisciplinary care in managing this rare but challenging condition.

## Acknowledgments

We would like to thank Editage (www.editage.jp) for English language editing.

## Author contributions

**Conceptualization:** Hiroki Hamamoto.

**Data curation:** Eiho Sawamura.

**Resources:** Eiho Sawamura, Hiroki Hamamoto.

**Supervision:** Hiroki Hamamoto.

**Writing – original draft:** Eiho Sawamura.

**Writing – review & editing:** Hiroki Hamamoto, Yusuke Suzuki, Toru Kuramoto, Takafumi Shima, Kazuya Kitada, Mitsuhiro Asakuma, Sang-Woong Lee.
